# Development of a method for estimating asari clam distribution by combining three-dimensional acoustic coring system and deep neural network

**DOI:** 10.1038/s41598-024-77893-7

**Published:** 2024-11-02

**Authors:** Tokimu Kadoi, Katsunori Mizuno, Shoichi Ishida, Shogo Onozato, Hirofumi Washiyama, Yohei Uehara, Yoshimoto Saito, Kazutoshi Okamoto, Shingo Sakamoto, Yusuke Sugimoto, Kei Terayama

**Affiliations:** 1 Graduate School of Medical Life Science, Yokohama City University, 1-7-29, Suehiro-cho, Tsurumi-ku, Yokohama, Kanagawa 230-0045 Japan; 2 Department of Environment Systems, Graduate School of Frontier Sciences, The University of Tokyo, Kashiwanoha, Kashiwa, Chiba 277-8561 Japan; 3 Shizuoka Prefectural Research Institute of Fishery and Ocean, 5005-3, Bentenjima, Maisaka-cho, Chūō-ku, Hamamatsu-shi, Shizuoka 431-0214 Japan; 4 Marine Open Innovation Institute, 2nd Floor, Shimizu Marine Building, 9-25, Hinode-cho, Shimizu-ku, Shizuoka-shi, Shizuoka, 424-0922 Japan; 5 Windy Network Corporation, 1-19-4, Higashi-Hongo, Shimoda-shi, Shizuoka, 415-0035 Japan; 6 RIKEN Center for Advanced Intelligence Project, 1-4-1, Nihonbashi, Chuo-ku, Tokyo, 103-0027 Japan; 7 MDX Research Center for Element Strategy, Tokyo Institute of Technology, 4259, Nagatsuta-cho, Midori-ku, Yokohama, Kanagawa 226-8501 Japan

**Keywords:** Marine biology, Ecology, Environmental sciences, Ocean sciences, Computational biology and bioinformatics

## Abstract

Developing non-contact, non-destructive monitoring methods for marine life is crucial for sustainable resource management. Recent monitoring technologies and machine learning analysis advancements have enhanced underwater image and acoustic data acquisition. Systems to obtain 3D acoustic data from beneath the seafloor are being developed; however, manual analysis of large 3D datasets is challenging. Therefore, an automatic method for analyzing benthic resource distribution is needed. This study developed a system to estimate benthic resource distribution non-destructively by combining high-precision habitat data acquisition using high-frequency ultrasonic waves and prediction models based on a 3D convolutional neural network (3D-CNN). The system estimated the distribution of asari clams (*Ruditapes philippinarum*) in Lake Hamana, Japan. Clam presence and count were successfully estimated in a voxel with an ROC-AUC of 0.9 and a macro-average ROC-AUC of 0.8, respectively. This system visualized clam distribution and estimated numbers, demonstrating its effectiveness for quantifying marine resources beneath the seafloor.

**Supplementary Information:**

The online version contains supplementary material available at 10.1038/s41598-024-77893-7.

## Introduction

The seafloor harbors numerous benthic organisms, which are indispensable as marine resources targeted by fisheries and for marine ecosystems and material cycles^1,2^. However, as most benthic organisms are always concealed in sediments, determining their population size and observing their behavior is difficult. Surveys are inevitably destructive, time-consuming, and costly. Conventional sampling methods cannot cover large areas, making it difficult to detect changes over time. This limitation is an impediment to sustainable management of sub-benthic resources and environment.

Benthic organisms such as asari clam (Japanese littleneck clam, *Ruditapes philippinarum*) have become a concern regarding stock management^3^. The asari clam is a bivalve that lives in the shallows of inner bays and is an important target fishery species. The harvest of asari clams in Japan has been on a downward trend, with a significant decrease from 160,000 tons in the 1980s to less than 10,000 tons since 2016. Various factors have been revealed as causes for this trend, including overfishing, feeding damage, disease and insect damage, and the prey environment^4^. Monitoring of clams has recently been conducted with the aim of sustainable fisheries management and resource conservation; however, this relies on manual digging and counting^5^. The current methods are very costly, and comprehensively monitoring the dynamics trends in the asari clam ecosystem over time is difficult.

Various monitoring methods have recently been developed by linking technological improvements in image and acoustic data acquisition with machine learning analysis, including deep learning^6–10^. For example, two-dimensional (2D) imaging of tracer particles^11^ and three-dimensional (3D) computed tomography (CT) imaging^12^ have been developed as data acquisition methods under the seafloor. Acoustic systems with various operating frequencies are commonly used to detect objects buried in seafloor sediments. For instance, wooden wrecks buried on the seafloor can be visualized using chirp signals with sweep pulses ranging from 1.5 to 13 kHz^13^. Recently, a new monitoring tool, a 3D acoustic coring system, has been developed and used to precisely survey buried roots of plants with an outer diameter of 3 to 5 cm using ultrasonic waves with a center frequency of 100 kHz^14^. In addition, high-frequency signals with a center frequency of 1 MHz have recently been used to survey small creatures of 3–5 cm, such as the clams^15^. Other deep learning analysis methods have been proposed for monitoring seafloor data, such as corals and seaweeds^6–8^. Furthermore, attempts have been made to classify organisms under the seafloor in the laboratory from data acquired with the 3D acoustic coring system^9^. However, such analysis methods need to be verified outside of the laboratory for the practical use of the 3D acoustics to estimate the distribution of targets in the obtained 3D data and the validation of such methods for benthic resource managements.

In this study, we developed a system to estimate the distribution of asari clams in a non-contact and non-destructive manner. Our system first acquires 3D benthic data containing clams using the 3D acoustic coring system. Then, the system predicts whether and how many clams are present in a local voxel region using a 3D convolutional neural network (3D-CNN)^16^, a method that has been used successfully in a wide range of tasks involving 3D data^10,17–22^. The proposed system can also estimate the distribution and number of targets by integrating the prediction results at multiple voxels within a certain region. To validate the proposed system, benthic organisms, such as clams and mussels, were obtained to verify the estimation. We also reported examples of the distribution visualization and estimation results of the count distribution within a certain region. Furthermore, we showed that Gradient-weighted Class Activation Mapping (Grad-CAM)^23^, a visual explanation method for interpretation in the prediction of neural network models, can be used to analyze and interpret regions that are important for the prediction of 3D data.

## Results and discussion

### Overview

**Fig. 1:**
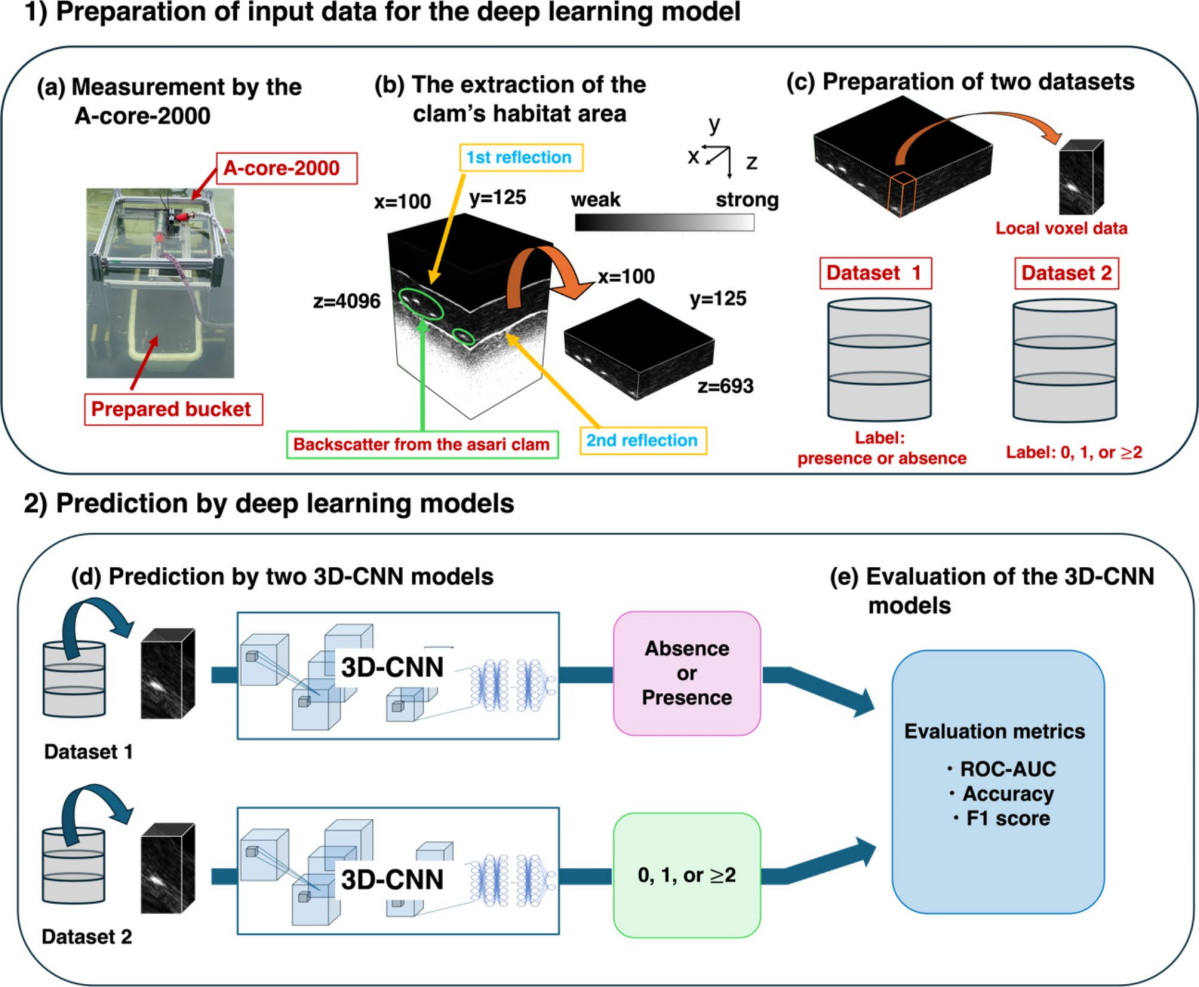
The workflow of the proposed system. This system is divided into two parts: 1) preparation of input data for the deep learning model and 2) prediction using the deep learning model. In 1), the A-core-2000^24^ was first used to measure the reflected waves from the subseafloor (**a**), extract the areas where clams are present (**b**), and create datasets on the presence/absence and the count of clams for training and evaluation of the deep learning model (**c**). In 2), the datasets created in (**c**) were used to train two independent 3D-CNN (3D convolutional neural network) models (**d**), and finally, the 3D-CNN models were evaluated using three evaluation metrics (**e**).

The workflow of this study is shown in Fig. 1. The proposed system was divided into two parts: 1) preparation of input data for the deep learning model and 2) prediction by two 3D-CNN models. The 3D-CNN models predicted the presence or absence of clams and the count of clams in the local voxel data.

## Model for classifying the presence or absence of clams

**Fig. 2:**
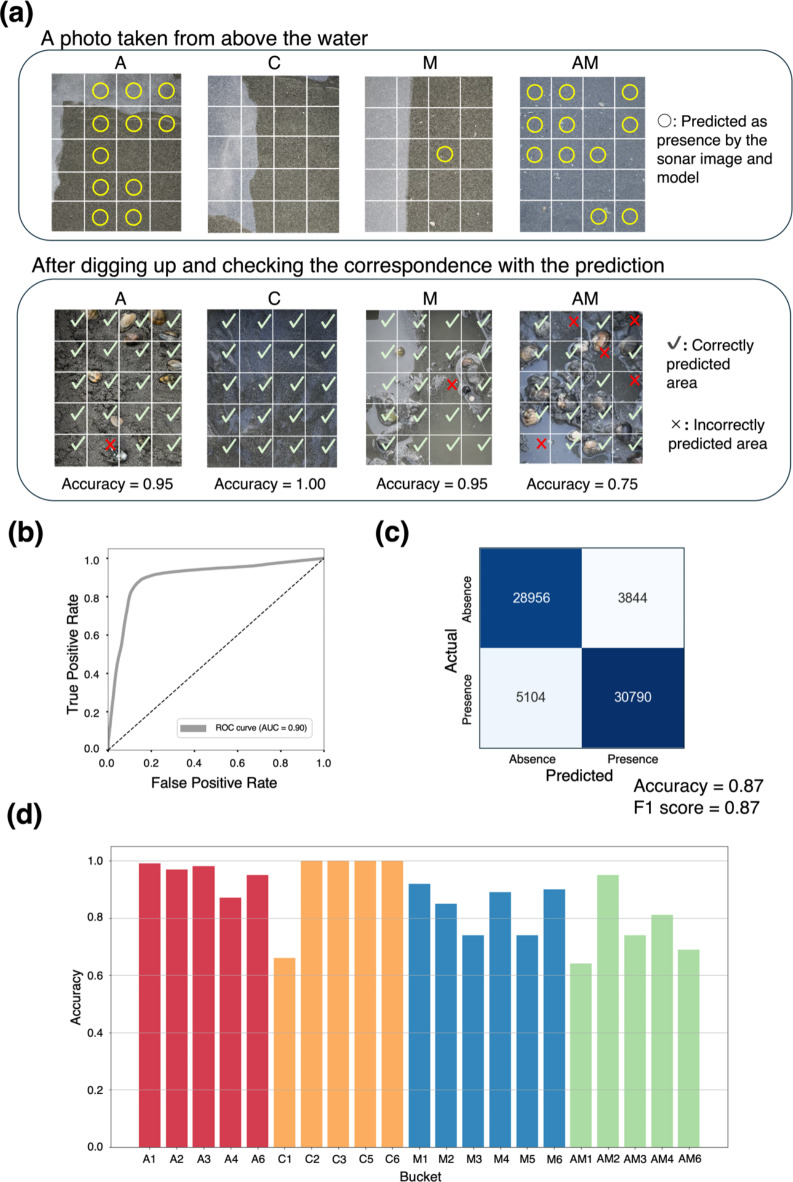
Prediction performances of the model for predicting the presence or absence of clams in a voxel. The model was trained and evaluated using Dataset 1 (presence or absence of clams). (**a**) Predicted distribution examples for each data type: control data containing sand only (C), data containing asari clams and sand (A), data containing a mixture of mussels and sand (M), and data containing a mixture of clams, mussels, and other materials (AM). Note that the presence or absence of clams is predicted from the corresponding voxel data, not from these photo images. The prediction is performed for the voxel corresponding to each square on these images. Regions marked with × indicate failed predictions, and regions marked with ✔ indicate successful predictions. (**b**) and (**c**) The prediction accuracy and the ROC curve for each voxel. (**d**) The prediction accuracy for each bucket.

First, we constructed the 3D-CNN model to discriminate the presence or absence of clams from the 3D data measured with the A-core-2000. The model training and evaluation were conducted using Stratified Group 5-fold cross-validation with the aforementioned data types A and C, including data types M containing mussels and sand and AM containing clams, mussels, and others (Fig. 2(a)). Figure 2(b) and (c) shows the model’s performance using ROC-AUC curves and confusion matrices. The prediction achieved an ROC-AUC of 0.90, an accuracy of 0.87, and an F1 score of 0.87. The detailed predictions for each data are shown in Fig. 2(d). Data type A containing only clams consistently exhibited high predictive accuracy, with accuracy exceeding 0.8 across all buckets. In contrast, AM and M showed several buckets with accuracy below 0.80, indicating lower overall accuracy than A. Additionally, most of the data in C showed high accuracy, except for C1, which had a value below 0.8. Predictions using only the intensity information of each voxel without 3D-CNN were also performed for comparison. Supplementary Fig. 1(a) and (b) shows the classification results using the mean and maximum intensity values for each voxel, respectively, with very low ROC-AUC values of 0.51 and 0.53. These results indicate that the presence or absence can be estimated with a certain level of accuracy using 3D-CNN.

**Fig. 3:**
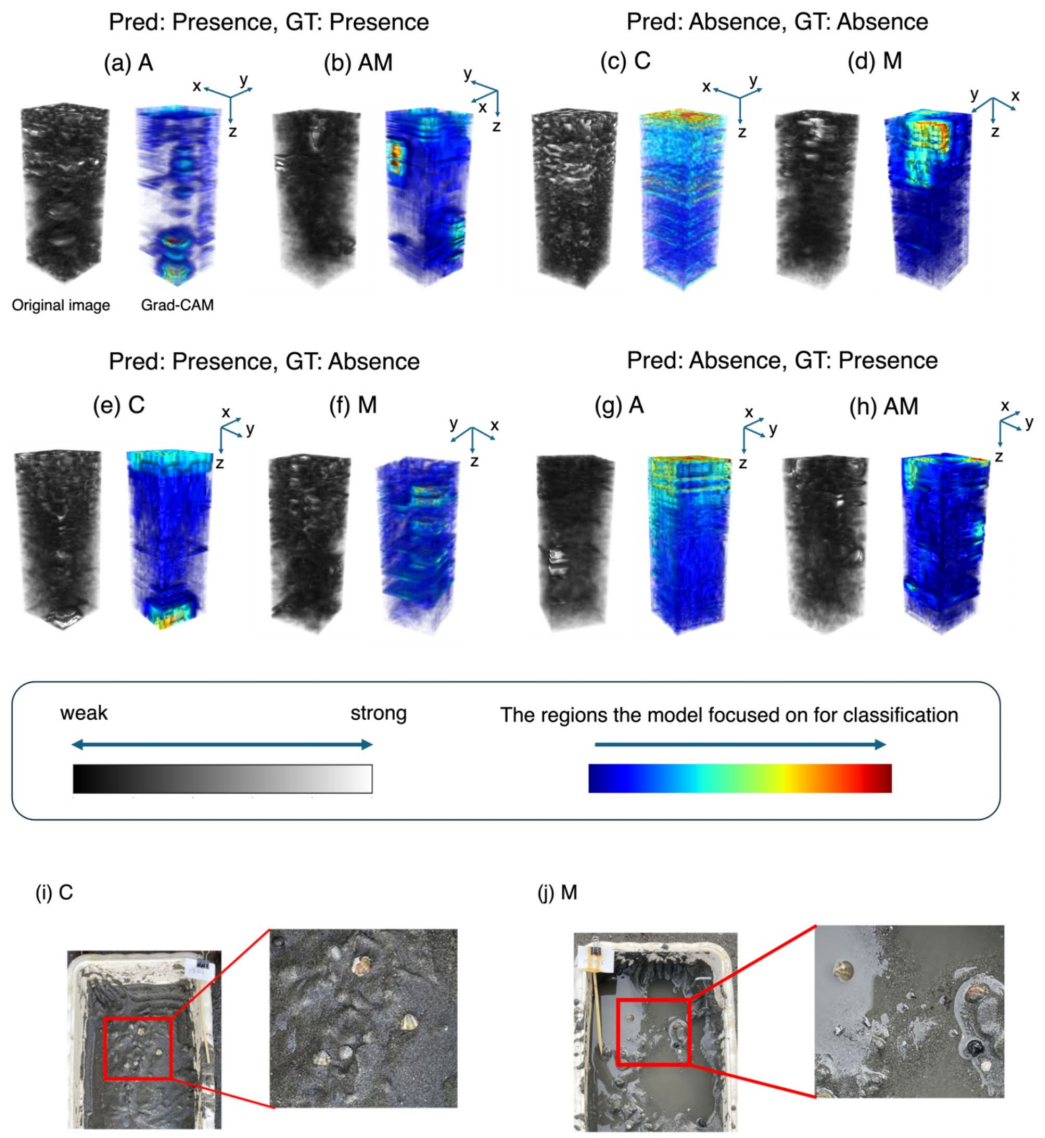
Visualized attention by the prediction model using Grad-CAM. The visualized data were averaged every 10 pixels in the z-direction. Pred indicates predicted results; GT indicates Grand Truth. Data type: control data containing sand only (C), data containing asari clams and sand (A), data containing a mixture of mussels and sand (M), and data containing a mixture of clams, mussels, and other materials (AM). (**a**) to (h) The original input data visualized in grayscale (left) and the corresponding heatmap calculated by Grad-CAM (right). (**a**) A data where both prediction and actual were “presence.” (**b**) AM data where both prediction and actual were “presence.” (**c**) C data where both prediction and actual were “absence.” (**d**) C data where both prediction and actual were “absence.” (**e**) C data where the actual was “absence” but predicted as “presence.” (**f**) M data where the actual was “absence” but predicted as “presence.” (**g**) A data where the actual was “presence” but predicted as “absence.” (**h**) AM data where the actual was “presence” but predicted as “absence.” (**i**) Excavation scene for C data where the actual was “absence” but predicted as “presence.” (**j**) Excavation scene for M data where the actual was “absence” but predicted as “presence”.

We used Grad-CAM^23^ to analyze where the prediction model focuses on in a 3D acoustic image to determine the presence or absence of clams. Grad-CAM enables the visualization of regions that constitute the basis for the predictions made by the model. Figure 3 shows the original images and the Grad-CAM visualizations averaged every 10 pixels in the z-direction. The upper rows of Fig. 3 show examples of successful predictions visualized by Grad-CAM. Figure 3(a) and (b) are cases in which clams are present in the voxel, and (c) and (d) are cases in which clams are absent. Figure 3(a) and (b) shows that when the model predicted the voxel as the presence of clams, it responded strongly to signals that appeared to be clams in relatively deep areas. In contrast, the model focused on shallow areas when predicting the voxel as the absence of clams. The lower row of Fig. 3 shows examples of failed predictions, whereas (e) and (f) represent cases where clams were predicted to be present even though there were no clams. Relatively strong signals were observed in the acoustic images; thus, the model responded to these signals and predicted the voxel as the presence of clams. As observed in (i) and (j) in Fig. 3, even C, which contained only sand, contained small clams and stones, which may have adversely affected the prediction. Although signals that appeared to be clams were present in the acoustic images and were focused by the model to some extent, the predictions were incorrect. These results suggest that although there remains room for improvement in accuracy, the prediction model focuses on signals that appear to be clams and the prediction of the presence of clams.

## Model for classifying the count of clams

**Fig. 4:**
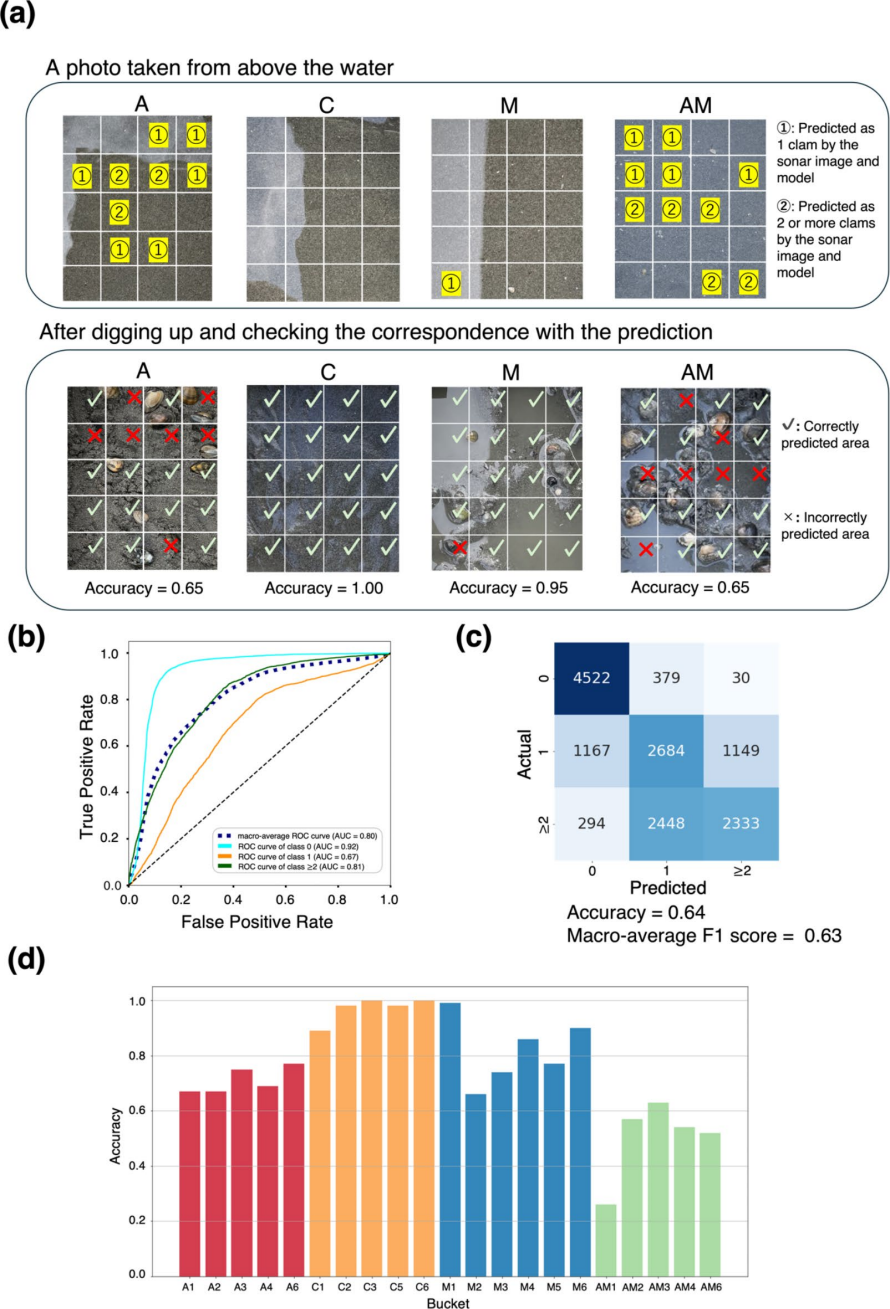
Prediction results of the model for estimating the clams count. The model was trained using Dataset 2 (labels: 0 clams, 1 clam, and 2 or more clams). (**a**) Examples of predictions for each data type: control data containing sand only (C), data containing asari clams and sand (A), data containing a mixture of mussels and sand (M), and data containing a mixture of clams, mussels, and other materials (AM). Regions marked with × indicate failed predictions, and regions marked with ✔ indicate successful predictions. (**b**) and (**c**) The prediction model’s performance for each voxel regarding ROC curves and confusion matrix. (**d**) The prediction accuracy for each bucket.

Next, the model for estimating the count of clams was trained and evaluated. The model training and evaluation were conducted through Stratified Group 5-fold cross-validation. The output was performed as a 3-class classification: “0”, “1”, and “2 or more.” Fig. 4(a) shows the prediction examples for each data type. Figure 4(b) and (c) shows the model’s performance using ROC-AUC curves and confusion matrix. The prediction achieved a macro-average ROC-AUC of 0.90, an accuracy of 0.64, and a macro-average F1 score of 0.64. The confusion matrix indicated that the discrimination accuracy of “1” and “2 or more” was lower than the prediction accuracy of “0”. This is probably because predicting the count of clams becomes difficult when the reflections overlap due to the high density of clams. The prediction results for each data type regarding accuracy are also shown in Fig. 4(d). The accuracy of data type C, which contained only sand, was high, whereas that of data types A and M was slightly low. The accuracy of data type AM, containing various objects other than clams, was further reduced. This result suggests that small stones or clams may make classification more difficult, as discussed in the model predicting presence.

## Estimating the distribution of clams

**Fig. 5:**
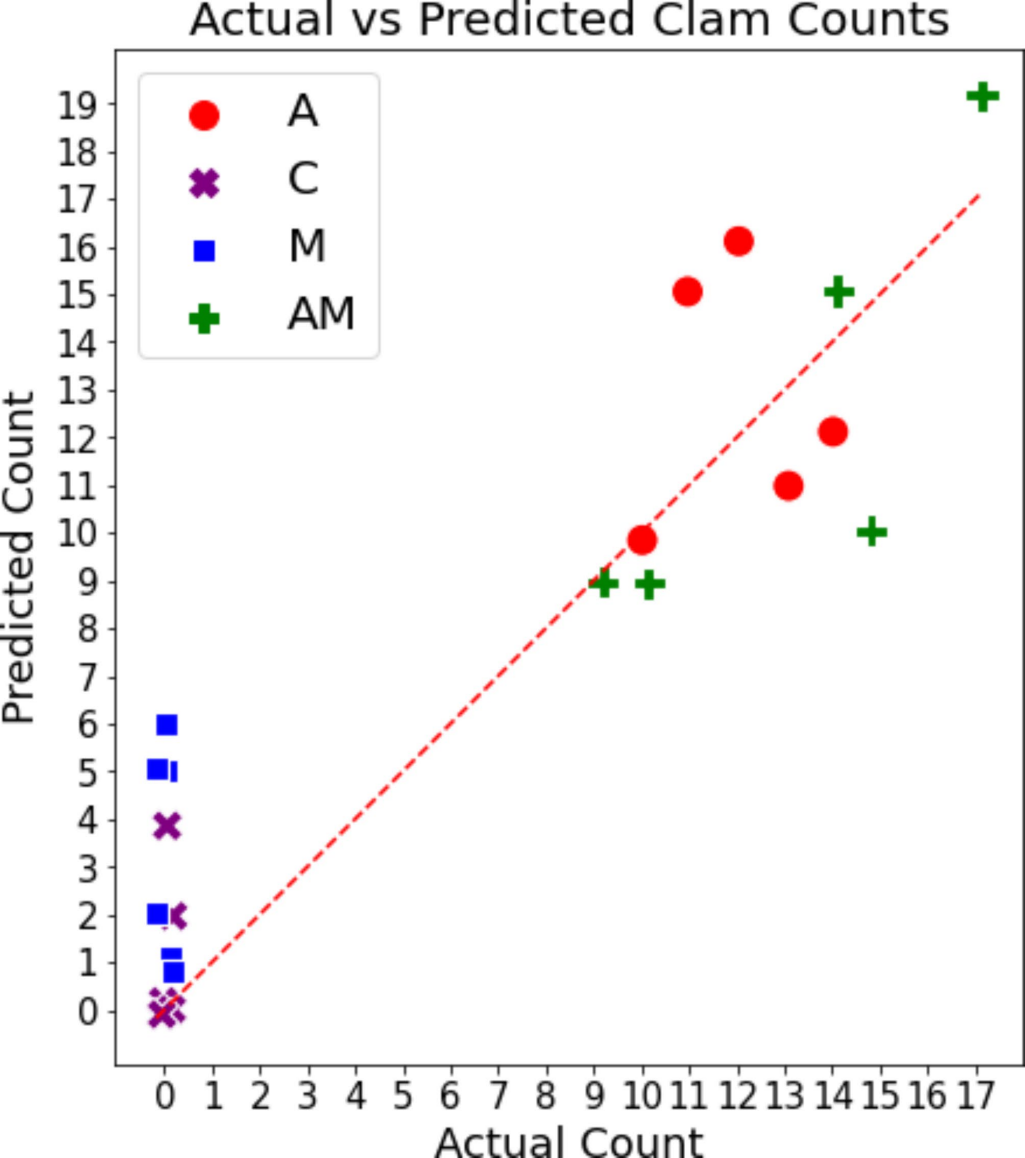
Estimated and actual count of clams per local region (bucket). The count of clams per bucket was calculated by integrating the results predicted by the model to estimate the count of clams. Data type: control data containing sand only (C), data containing asari clams and sand (A), data containing a mixture of mussels and sand (M), and data containing a mixture of clams, mussels, and other materials (AM).

The proposed method enabled us to estimate the distribution of clams (Fig. 2(a) and 4(a)) because the trained models predicted the presence and count of clams in each voxel. Furthermore, the count of clams within a certain region containing multiple voxels was estimated by integrating the prediction results. Here, we evaluated the accuracy of estimating the clams contained within each bucket’s region, consisting of 20 voxels (Fig. 5). The estimated and measured counts for each bucket are listed in Supplementary Table 1. The overall correlation coefficient was 0.92, and the correlation coefficient for only the A and AM data was 0.68, confirming the correlations of the clam counts. The mean absolute error (MAE) and mean relative error (MRE) for each data type are presented in Table 1. The MRE values were 0.20 for A and 0.12 for AM, indicating that the estimation is possible with an error of approximately 10–20%. These results suggest that the count of clams in each region can also be estimated, although with some error.

**Table 1:** MAE (mean absolute error) and MRE (mean relative error) for predicting the count of clams for each data type: control data containing sand only (C), data containing asari clams and sand (A), data containing a mixture of mussels and sand (M), and data containing a mixture of clams, mussels, and other materials (AM). The count of clams was estimated for each bucket using the prediction model of the count of clams. The MAE in the count of clams was calculated for each data type. MRE was calculated only for A and AM because relative absolute error cannot be calculated when the count of clams is zero.

	A	C	M	AM
MAE	2.4	1.2	3.3	1.8
MRE	0.20	-	-	0.12

## Conclusion

In this study, we prepared and measured benthic data, including clams, to validate the resource survey method using the three-dimensional acoustic coring system. Using the measured data, two prediction models based on 3D-CNN were trained and evaluated to estimate the presence and count of clams. We successfully estimated the presence or absence of clams in each voxel with an ROC-AUC of approximately 0.9 and the count of clams in local areas with a macro-average ROC-AUC of 0.8. The count of clams within a certain area (bucket) was confirmed to be estimated by MAE values of 0.12 or 0.20. These results indicate the potential of the proposed method for estimating benthic resources.

Unlike conventional sampling methods that involve drilling and disturbance of the marine environment, this method does not require physical interference with the ecosystem, thus minimizing potential harm to other marine species and maintaining habitat integrity. This reduced environmental impact is a significant advantage over traditional methods and would be a more sustainable option for resource surveys. Additionally, while traditional methods have often difficulties grasping spatial distribution and temporal changes, the approach proposed in this study allows for the observation of these aspects.

However, this study has the potential for improvement. First, the prediction accuracy tended to decrease when factors other than clams were introduced, such as AM. To solve this problem, it is beneficial to prepare a larger amount of various data and to use more advanced neural networks, such as vision transformers, to process voxel data for learning. Because the measurement speed of data by A-core-2000 is slow and the measurement area is relatively narrow, considerably faster measuring devices (array sonar system) are needed. As this study was conducted in a relatively controlled environment, verification in the outside field is desired in the future.

### Methods

#### Data preparation and measurement using the acoustic coring system

##### Preparation of benthic data containing clams

Benthic data, including clams, were obtained from a portable pool at the Hamanako Branch, Shizuoka Prefectural Research Institute of Fishery, Shizuoka Prefecture, Japan. Four types of benthic data were prepared to verify whether estimation could be performed for various types of data: control data containing sand only (C), data containing asari clams and sand (A), data containing a mixture of mussels and sand (M), and data containing a mixture of clams, mussels, and other materials (AM). Each benthic data was prepared by placing sand and mussels from Lake Hamana in a rectangular bucket with internal dimensions of 580 × 155 × 200 mm. Six buckets were prepared for each data type. For the A and AM data, 40 live clams from the nearby Mikawa Bay were placed in each bucket three days before the measurement date and stored until the measurement date.

##### Measurement using the acoustic coring system

A convergent ultrasonic sensor, the A-core-2000^24^ was used to observe benthic data in the prepared buckets (Fig. 1(a)). The bucket’s 250 × 200 mm area was scanned at 2 mm intervals while continuously irradiated with ultrasound, and the reflected sound wave was measured and recorded. Square pulse with a central frequency of 500 kHz was generated by a pulsar receiver in the acoustic unit and applied to the focus probe. The pulse repetition interval was 200 ms. The distance of focal point, beam width, and focal depth were around 70 mm, 4 mm, and 30 mm, respectively. The median grain size of sands which we used in this study was 2.6 $\:\times\:$ 10^−4^ and thus the attenuation coefficient was estimated 10^2^ dB/m according to the analysis in the previous study^24^. In this study, the absorption attenuation was considered dominant because the ratio $\:k$d ($\:\text{k}\:=\:\text{w}\text{a}\text{v}\text{e}\text{n}\text{u}\text{m}\text{b}\text{e}\text{r}$, $\:\text{d}\:=\:\text{g}\text{r}\text{a}\text{i}\text{n}\:\text{s}\text{i}\text{z}\text{e}$) was 8.6 $\:\times\:\:$ 10^−2^ and this value is quite small (kd$\:\ll\:$1)^25^. Envelope processing was performed on the recorded waveform data. The acoustic image constructed by the Viewer^24^ showed that the reflection from the clams was between reflections from the soil surface and reflection from the soil surface again (the 2nd reflection in Fig. 1(b)). In this study, the range of analysis was defined as the area between the first and second reflections (multiple reflections) from the sediment surface. The total reflection intensity in the XY plane was calculated for each z-coordinate in the 3D data of the acquired reflection intensity, the region of clam presence was extracted, and the size was standardized for each bucket (Sl). Therefore, the 3D data of reflectance intensity measured at 125 × 100 × 693 points in each bucket were used in this study.

##### Manual identification of positions of asari clams

The positions of asari clams in the measured data were identified to evaluate the prediction models. First, x and y coordinates, the candidate positions of asari clams in the horizontal direction, were manually obtained from the reflection intensity data. Next, the positions of the asari clams were identified by comparing them with the image data of the dug clams taken at the time of measurement. The backscatter of the clams was measured, and the center position of the backscatter was defined as the clam position. The data on the positions of the clams were recorded in Supplementary Tables 2 and 3. The data in A5, C4, and AM5 were excluded from the data set because mechanical issues with the probe resulted in excessive noise, rendering the measurements inaccurate.

##### Preparation of datasets for model training and evaluation

Datasets 1 and 2 were created to construct models to predict the presence or absence of clams in a local voxel and the count of clams in a local voxel. First, the data obtained from each bucket was divided into 25 × 25 pixels, shifting every 1 pixel in the horizontal direction, resulting in 124,689 local voxel data, each with size 25 × 25 × 693 pixels (Fig. 1(b)). Here, backscatter data of clams could be included in the boundary of the divided data. Therefore, if the position of a clam recorded above was included in a local voxel or the shortest distance of the position from a local voxel was less than 11 pixels, the local voxel data was regarded as including the clam. Based on the positions of clams identified in the above Section, for Dataset 1, a label of “Absence” was assigned if no clams were included in a local voxel data, and “Presence” was assigned if at least one clam was included in a local voxel data. The number of labeled data is presented in Table 2; for C and M, all data were labeled as “absence” because no clams were included in the buckets. Based on the positions of clams identified in the above Sect. 3.1.3, for Dataset 2, the count of clams was assigned as a label (Table 3). The number of data containing three or more clams in one local voxel was very small (1,137); therefore, data containing three or more clams were treated as data containing two or more clams. Datasets 1 and 2 were created by randomly extracting data from the 124,689 local voxel data, each with a size of 25 × 25 × 693 pixels; thus, the number of data for each label was approximately the same.

**Table 2:** Dataset regarding the presence or absence of clams (Dataset 1). Data type: control data containing sand only (C), data containing asari clams and sand (A), data containing a mixture of mussels and sand (M), and data containing a mixture of clams, mussels, and other materials (AM).

	Absence	Presence
A	2,125	16,717
C	13,000	0
M	15,600	0
AM	2,075	19,177
Total	32,800	35,894

**Table 3:** Dataset on the count of clams (Dataset 2). Data type: control data containing sand only (C), data containing asari clams and sand (A), data containing a mixture of mussels and sand (M), and data containing a mixture of clams, mussels, and other materials (AM).

	0	1	$\:\ge\:$2
A	1,147	2,500	1,939
C	1,250	0	0
M	1,500	0	0
AM	1,034	2,500	3,136
Total	4,931	5,000	5,075

##### Classification using deep neural networks

**Fig. 6:**
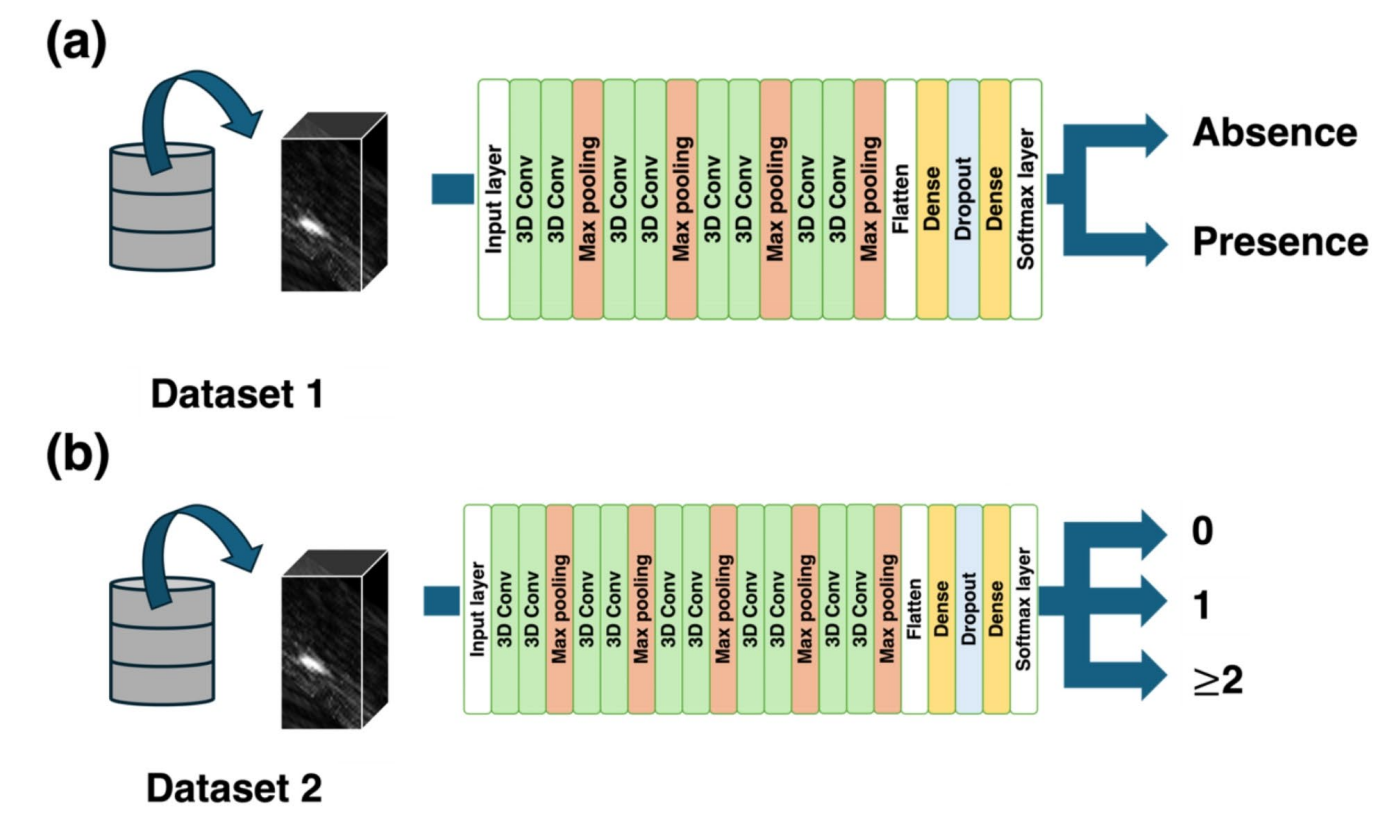
Overview of predictions using 3D-CNN (3D convolutional neural network). The 3D-CNN models predict (**a**) the presence/absence of clams and (**b**) the count of clams (0, 1, and “2 or more”) from the input of 3D acoustic data. For training and evaluation of the models, Dataset 1 and 2, shown in Fig. 1, were used for the predictions (**a**) and (**b**), respectively.

In this study, we employed the 3D-CNN^16^ to develop the prediction models. 3D-CNN has demonstrated success in various tasks involving 3D data, such as classification, detection, and segmentation of medical images^10,17–20^, as well as action recognition^21,22^. It mainly consists of convolutional, pooling, and fully connected layers. We modified the 3D-CNN developed in our previous study^9^ to build two independent models: one for classifying the presence or absence of clams and another for classifying the count of clams. In the model for classifying the presence or absence of clams, we used Dataset 1, containing information on the presence or absence of clams as input and obtained outputs of either ‘Absence’ or ‘Presence’ (Fig. 6). The model employed the Adam optimizer, with a batch size of three and a learning rate of 10^−6^, and was trained for a maximum of 50 epochs, stopping if there was no improvement for 10 epochs. For the model classifying the count of clams, we used Dataset 2, containing information on the count of clams as input, and obtained outputs of ‘0’, ‘1’, or ‘2 or more clams’ (Fig. 6). The model employed the Adam optimizer, with a batch size of three and a learning rate of 10^−6^, and was trained for a maximum of 30 epochs, stopping if there was no improvement for 10 epochs.

In this study, TensorFlow (version 2.4.1)^26^ was used to construct two 3D-CNN models, and the performance of the models was evaluated using Stratified Group 5-fold cross-validation^27^. The groups represented each bucket. Additionally, the dataset was divided into training (60%), validation (20%), and test (20%) sets.

## Evaluation metrics of the prediction model

Three evaluation metrics were employed to evaluate the trained models: accuracy, F1-score, and the area under the receiver operating characteristic curve (ROC-AUC). Accuracy represents the proportion of correctly predicted data, and this metric is the most basic measure to evaluate the overall performance of a classification model. F1-score is defined as the harmonic mean of precision and recall:

$$\:F1-score=\frac{2\cdot\:precision\cdot\:recall}{precision+recall}$$.

The precision indicates the percentage of data predicted to be positive that is actually positive, and the recall indicates the percentage of actual positive data that is predicted to be positive. The F1 score was employed to evaluate the balance between precision and recall. ROC-AUC refers to the area under the ROC curve, which plots the true-positive rate against the false-positive rate as the classification threshold varies. ROC-AUC was employed to evaluate how well the model was able to detect the presence of clams while allowing for certain errors, balancing true positive and false positive rates at different thresholds. Each metric ranges from 0 to 1, with higher values indicating better model performance.

Furthermore, to evaluate the model for classifying the count of clams, we used macro-average ROC-AUC and F1-score, which are the averages of the ROC-AUC and F1-score calculated for each class. Macro-average refers to the method of computing the ROC-AUC and F1-score for each class individually and then averaging these scores. This metric considers the performance of each class equally and allows for a balanced evaluation of the overall performance, even in cases with class imbalance.

## Electronic Supplementary Material

Below is the link to the electronic supplementary material.

Supplementary Material 1

## Data Availability

The acoustic voxel data have been archived on Zenodo. You can access the datasets using the following links: https://zenodo.org/records/13381836, https://zenodo.org/records/13377864, https://zenodo.org/records/13381893.
