# Supplementary material for: Development of a method for estimating asari clam distribution by combining three-dimensional acoustic coring system and deep neural network
Source: Sci Rep. 2024 Nov 2;14:26467. doi: 10.1038/s41598-024-77893-7 (PMC11531588; doi:10.1038/s41598-024-77893-7)
Supplement: Supplementary file 1 — Supplementary Material 1 [file 41598_2024_77893_MOESM1_ESM.pdf]

Supporting Information for

Development of a method for estimating asari clam distribution by combining three-dimensional acoustic coring system and deep neural network

Tokimu Kadoi[1], Katsunori Mizuno[2*], Shoichi Ishida[1], Shogo Onozato[2], Hirofumi Washiyama[3], Yohei Uehara[3], Yoshimoto Saito[4], Kazutoshi Okamoto[4], Shingo Sakamoto[5], Yusuke Sugimoto[5] and Kei Terayama[1,6,7*]

[1]Graduate School of Medical Life Science, Yokohama City University, 1-7-29, Suehiro-cho, Tsurumi-ku, Yokohama, 230-0045, Kanagawa, Japan.

[2]Department of Environment Systems, Graduate School of Frontier Sciences, The University of Tokyo, Kashiwanoha, Kashiwa, Chiba, 277-8561, Japan.

[3]Shizuoka Prefectural Research Institute of Fishery and Ocean, 5005-3, Bentenjima, Maisaka-cho, Chūō-ku, Hamamatsu-shi, Shizuoka, 431-0214, Japan.

[4]Marine Open Innovation Institute, 2nd Floor, Shimizu Marine Building, 9-25, Hinode-cho, Shimizu-ku, Shizuoka-shi, Shizuoka, 424-0922, Japan.

[5]Windy Network Corporation, 1-19-4, Higashi-Hongo, Shimoda-shi, Shizuoka, 415-0035, Japan.

[6]RIKEN Center for Advanced Intelligence Project, 1-4-1, Nihonbashi, Chuo-ku, 103-0027, Tokyo, Japan.

[7]MDX Research Center for Element Strategy, Tokyo Institute of Technology, 4259, Nagatsuta-cho, Midori-ku, Yokohama, 226-8501, Kanagawa, Japan.

*Corresponding author(s). E-mail(s): kmizuno@edu.k.u-tokyo.ac.jp and terayama@yokohama-cu.ac.jp

Extraction of the habitat of clams

The total value of reflection intensity on the xy plane at each z-coordinate was calculated. Next, after examining the positions of the 1st reflection and the 2nd reflection for each bucket, the 1st reflection was in the range of $800 \leqq z < 1600$ and the 2nd reflection was in the range of $1600 \leqq z \leqq 3000$. Therefore, to extract the area between the 1st and 2nd reflection, for each bucket, the coordinate of the peak Z in the range of $800 \leqq z < 1600$ plus 50 (Z1) and the coordinate of the peak Z in the range of $1600 \leqq z \leqq 3000$ minus 100 (Z2) were calculated. Then, Z2-Z1 was computed. Finally, the values of Z2-Z1 were resized to match the minimum value. Consequently, the three-dimensional data of reflectance intensity measured at 125 × 100 × 693 points in each bucket were used in this study.

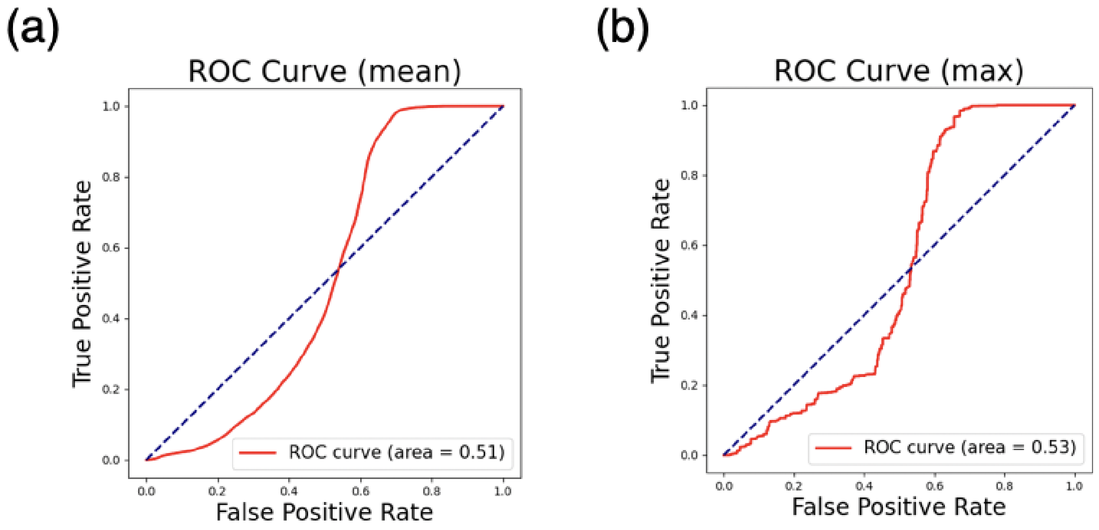

SI Figure 1. Performance of the prediction of the presence or absence of clams in a voxel based solely on reflection intensity. (a) ROC curve for predicting the presence or absence of clams in a voxel based on the average reflection intensity. (b) ROC curve for predicting the presence or absence of clams in a voxel based on the maximum reflection intensity.

SI Table 1. The number of clams present in the surveyed area and the predicted number of clams by a model designed to forecast their quantity, including the prediction error. For the prediction, clams with 2 or more voxels were counted as 2.

	Actual	Predict	Absolute error
A1	14	12	2
A2	13	11	2
A3	12	16	4
A4	10	10	0
A6	11	15	4
C1	0	4	4
C2	0	0	0
C3	0	0	0
C5	0	2	2
C6	0	0	0
M1	0	1	1
M2	0	6	6
M3	0	5	5
M4	0	1	1
M5	0	5	5
M6	0	2	2
AM1	15	10	5
AM2	17	19	2
AM3	14	15	1
AM4	10	9	1
AM6	9	9	0

SI Table 2. Position of reflection from clams in bucket A.

	x	y		x	y
A1	40	4	A4	16	5
	23	4		113	1
	31	7		87	1
	17	13		80	19
	8	15		8	17
	98	30		106	50
	39	35		44	49
	7	44		117	78
	6	61		35	75
	55	61		55	92
	79	62			
	54	69			
	38	73			
	122	45			
A2	34	6	A6	27	5
	58	18		70	11
	5	23		66	30
	92	31		119	36
	102	44		62	48
	17	54		89	56
	80	64		9	54
	43	96		32	59
	72	97		123	62
	25	98		108	78
	99	76		80	66
	3	97			
	114	94			
A3	22	13			
	38	11			
	44	35			
	63	44			
	88	42			
	39	52			
	52	90			
	94	97			
	9	97			
	114	98			
	72	16			
	110	79			

SI Table 3. Position of reflection from clams in bucket AM.

	x	y		x	y
AM1	109	4	AM4	104	4
	79	15		83	19
	37	14		47	19
	44	23		24	24
	9	25		69	27
	104	24		112	39
	97	31		74	57
	61	42		72	70
	123	51		31	96
	59	52		100	98
	84	53			
	61	61			
	99	88			
	76	83			
	52	98			
AM2	89	5	AM6	95	2
	100	13		48	22
	64	19		118	38
	114	21		68	52
	23	23		39	51
	105	34		7	68
	46	37		48	66
	104	52		83	60
	47	61		119	96
	83	65			
	55	72			
	23	77			
	123	72			
	94	86			
	117	93			
	36	92			
	104	98			
AM3	108	5			
	121	2			
	73	3			
	32	6			
	58	38			
	26	35			
	31	58			
	68	70			
	53	95			
	27	99			
	103	98			
	115	47			
	115	31			
	17	80			
